# Hybrid polaritonic switch with light-controlled Rabi splitting in molecular plasmonic system

**DOI:** 10.1039/d5na01061f

**Published:** 2026-04-17

**Authors:** Bill Brook Shurtleff, Ron-Marco Friedrich, Thomas Strunskus, Mady Elbahri, Franz Faupel

**Affiliations:** a Chair for Multicomponent Materials, Department of Materials Science, Kiel University Kaiserstrasse 2 Kiel 24143 Germany ff@tf.uni-kiel.de; b Department of Chemistry and Materials Science, Aalto University Kemistintie 1 Aalto 00076 Finland mady.elbahri@aalto.fi

## Abstract

Polaritonic switches and light control of strong coupling in molecular plasmonic systems are of fundamental and technological interest. Here, we show that coupling strength is made tunable in a photoswitchable plasmonic cavity formed at the interface between a metal and a chromophore-containing polymer. This setup is much simpler than approaches based on individual quantum emitters in high finesse optical cavities. We demonstrate reversible photoswitchable in *operando* Rabi splitting of ∼600 meV, as high as ∼29% of the molecular transition energy, and control of the coupling strength *via* irradiation time and chromophore concentration. The experimental results are confirmed by transfer matrix simulations with losses inherently included *via* the experimentally measured dielectric functions.

## Abstract 2

Reversible light-controlled Rabi splitting is achieved in a molecular plasmonic system, with coupling strength tuned by the concentration and photo-isomerization of embedded chromophores.

## Graphical abstract

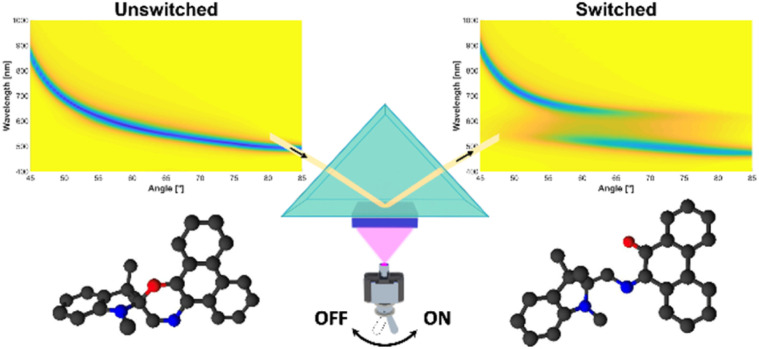

## Introduction

Resonance in optical systems is omnipresent, their oscillations sending and receiving electromagnetic waves all around us. When two oscillating systems approach each other, their emitted radiation can interfere and their wavefunctions coherently couple to form a polaritonic wavefunction. Polaritons are hybrid states, a superposition of light and matter where the coupling strength exceeds the damping rate of the system. A straightforward way to strongly couple oscillators can occur between molecular transitions and an optical mode. Strongly coupled oscillators range from dye/aggregate molecule excitons,^1–3^ cavity modes in Fabry-Pérot etalons^4^ to distributed Bragg reflectors,^5^ microcavities^6^ and nanocavities,^7,8^ quantum dot excitons,^9^ and plasmon–exciton systems.^10^ In molecular plasmonic systems, strong coupling has been demonstrated with surface plasmon polaritons (SPPs),^11^ lattice plasmons,^12^ localized surface plasmon resonances,^13^ and molecules (from one to many) coupled to a single plasmonic cavity^14^ or structure. Such polaritonic states in molecular plasmonic systems have been important not only for investigations of light–matter interactions but also for a host of promising applications, such as quantum computing,^15^ controlling chemical reactions^16^ and biomedical applications.^17^

Few attempts have been made to switch such coupling,^4^ particularly in plasmonic metal thin films with their attendant high intrinsic losses.^18^ Nonetheless, plasmonic and Rabi splitting switches in molecular photonics and plasmonics have been introduced based on the reflection mode.^19–22^ Photoswitching of strong plasmon–exciton coupling was also achieved by dynamic molecular aggregation, integrating plasmonic arrays in a microfluidic device.^23^ Moreover, in addition to light, other stimuli were employed, *e.g.* pH and temperature.^24^

**Fig. 1:**
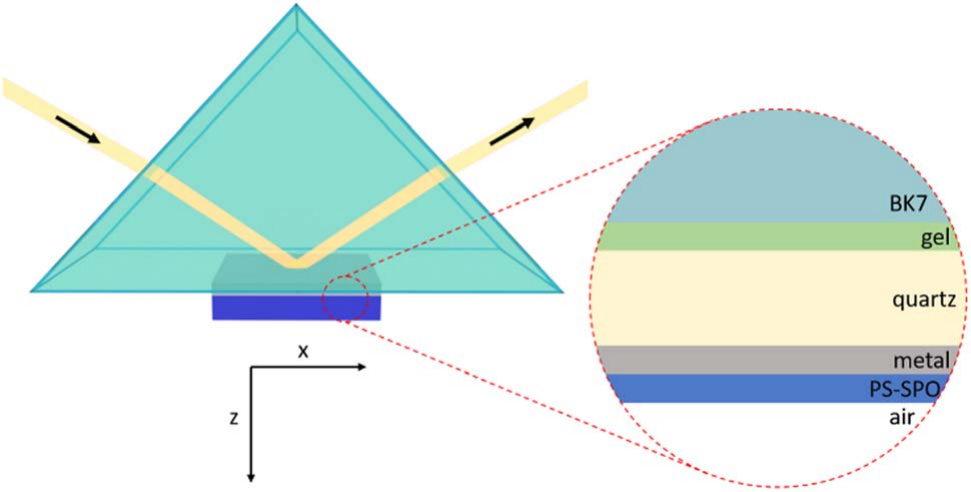
Schematic diagram of Kretschmann configuration for measurement of dispersion relations.

While such a concept can function as a starting point, a straightforward way to tailor and dynamically control the coupling regime of a switchable plasmonic cavity has not yet been introduced. Here, we show that coupling strength is made tunable by controlling the dielectric properties of a photoswitchable plasmonic cavity bounded on one side by the interface between a metal and a chromophore-containing polymer and on the other side by the evanescence of fields extending into the dielectric layer. This setup (see Fig. 1) is much simpler, and hence more attractive for practical applications, than approaches used before, *e.g.* based on individual quantum emitters in high finesse optical cavities. For instance, additional degrees of freedom to switch and control the coupling regime are gained through controlling the resonance frequency, type of metal, effective mode volume, illumination duration and molecular concentration. The off/on switching of the Rabi splitting presented here offers the ability to change a physical property of an optical system remotely, along with the high contrast of the avoided crossing. The hybrid polaritonic switching with light-controlled Rabi splitting is shown experimentally and confirmed by simulations, which proved indispensable for designing the system.

## Materials and methods

### Theoretical background

Although our approach involves highly lossy plasmons, it was inspired by the well-established physics of cavity quantum electrodynamics with negligible dissipation. There, the strong coupling strength *g* in a nanophotonic cavity as given in eqn (1) or (2) simply reads:^25^1
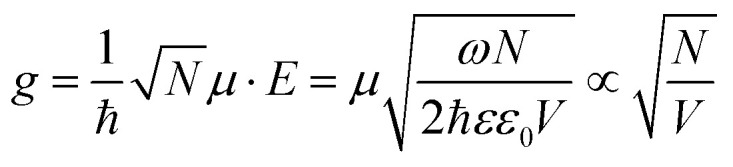


or2
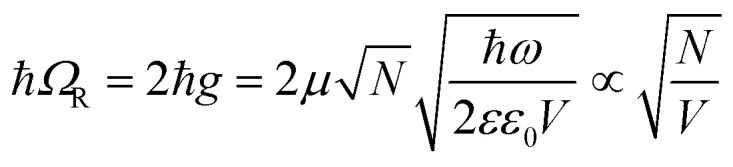
Here *µ* is the dipole moment of *N* identical emitters, *E* the cavity electric field experienced uniformly by the emitters, *ω* the angular frequency of the cavity light mode, *V* the mode volume for the cavity, and *ε* relative and *ε*_0_ vacuum permittivities; in eqn (2), *Ω*_R_ the Rabi splitting.

The strength of the coupling between matter and light can be tailored and controlled in several ways: by increasing *µ*, the oscillator strength of the matter part, or *N*, the number of coupled molecules in the system; by adjusting the resonance *ω* and material choice for *ε*; by confining *V* of the electromagnetic mode into a smaller optical cavity volume. Plasmonics offers just such a reduction in the mode volume *V* due to the confinement and enhancement of the field in plasmonic structures.

The first equality in eqn (1) holds for those interactions between light and matter where the dipole approximation applies, *i.e.*, the wavelength of the electromagnetic field is much greater than the size of the emitters, an approximation that applies here. The second equality in eqn (1) only holds when all *N* emitters are aligned in the uniform electric field, a situation not given in the Kretschmann setup (see Fig. 1) where the evanescence of the electromagnetic field assures a non-uniform electric field and the emitters are not expected to be well-aligned but instead randomly oriented in the dielectric matrix. In addition, the choice for the mode volume *V* becomes unclear in the open cavity of a Kretschmann configuration, where one “mirror” of the metal layer and the evanescence of the SPP-generated field form the cavity. What should be chosen as the volume for the mode? And does the ohmic loss in the metal layer render the model unusable?

Such questions have been raised in the literature^26^ as to what extent such theoretical models based on a two-level system or a collection of two-level systems in a non-radiative cavity with low losses and only one mode can apply to real systems where a radiative cavity may have higher losses and more than one mode. Questions still linger in the community whether equations developed for describing quantum two-level systems can be applied to systems with excitons acting collectively when interacting with the cavity mode(s) of hard-to-define mode volumes. In general, definitions of the mode volume *V* and the number of emitters *N* may become difficult depending on the experimental setup.

However, a completely *ab initio* quantum description of photon–emitter coupling between emitters and propagating surface plasmons on two-dimensional metal surfaces has been published.^27^ With this, the findings still reflect the √(*N*/*V*) proportionality relationship for the Rabi splitting, albeit without the difficulties of defining *N* and *V*.

For the work presented here, the real part of the refractive index *n* represents the important and essential parameter for the confinement, in addition to the evanescence of the field. An increase in *n* reduces the volume cavity further by reducing electromagnetic wavelengths, hence enhancing the coupling strength in a molecular plasmonic system.

The dependence of surface plasmon polariton confinement on the dielectric permittivity at a metal–dielectric interface is well established in the plasmonics literature. It provides a solid physical basis for the qualitative mode-volume argument used here, in line with the non-uniqueness of mode volume in open plasmonic systems discussed by Tserkezis *et al.*^26^ and the established dependence of SPP confinement on dielectric permittivity reviewed by Zayats *et al.*^28^

### Why photoswitchable molecules?

**Fig. 2:**
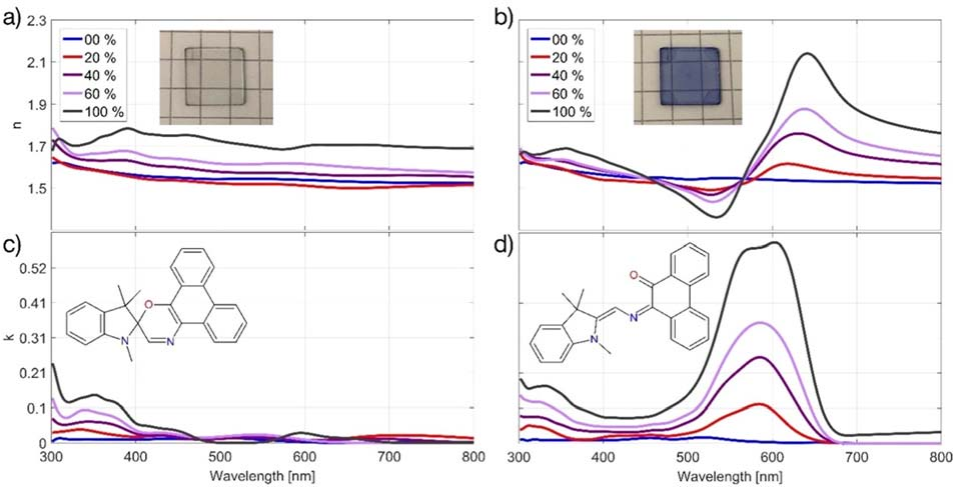
Complex index of refraction values of *n* (a and b) and *k* (c and d) for unswitched (a and c) and switched (b and d) thin film samples of a PS matrix with differing weight percent amounts of SPO mixed in (see legends).

With photoswitchable molecules of spirooxazine (SPO) embedded in a dielectric polystyrene (PS) matrix, *n* can be tailored not only by switching the molecules but also by adjusting their concentration (Fig. 2a). For the unswitched molecules, normal dispersion is evident in Fig. 2a with the real part of the refractive index *n* peaking close to 400 nm. Once switched, anomalous dispersion near 600 nm is apparent in Fig. 2b. Collectively excited states of the conjugated merocyanine (MC) molecules in proximity couple when oscillating coherently and act as artificial plasmonic dipoles.^22,29^ The broadening and asymmetry of the resonance in the switched composite PS-SPO (Fig. 2d) is well known (see, *e.g.*, ref. 29 and 30) and can be viewed as Davydov splitting,^31^ the interaction of degenerate dipoles due to their relative orientation and proximity, although direct imaging of the molecular aggregation is challenging and was not the focus of the present study.

Inclusion of photoswitchable molecular emitters in a plasmonic Kretschmann cavity (see Fig. 1) while controlling the optical properties enables photoswitchable Rabi splitting. When matter with a well-defined electronic transition is placed proximate to a plasmonic environment with a frequency that coincides with the matter's transition energy, they may interact and couple. If the energy exchange rate is faster than any dissipation in the system, the interaction will give rise to polaritonic states indicative of a strong coupling regime. Here, the experiments resulted in ultrastrong coupling involving lossy plasmons on silver in a classical Kretschmann configuration, shown in Fig. 1, with a cavity loaded with 60 wt% SPO to reduce the plasmonic cavity mode volume *V* even more upon switching.

## Experimental

For the experimental implementation of the Kretschmann configuration, clean quartz squares (1 cm^2^, 525 µm thick) were first covered with a layer of Ag in a commercial plasma-magnetron sputter chamber, a Balzers SCD 050 Sputter Coater. Layer thickness was verified on a spectroscopic ellipsometer (M-2000UI from J.A. Woollam Co., Inc.) and CompleteEASE software. Polystyrene (Roth, 9151.1) and 1,3-dihydro-1,3,3-trimethylspiro[2*H*-indole-2,3-[3*H*] phenanthr[9,10-*b*](1,4) oxazine] (here, SPO) (Chemical Point, CAS 119980-36-8) mixtures of the desired weight percent of SPO were prepared by dilution in toluene (Roth, 4445.1, UV-IR grade). PS-SPO solutions were deposited onto the metal layer with a commercial spin coater (Laurell, Model WS-650MZ-23NPPB) as follows: 50 µL solution pipetted onto non-spinning samples, spun up to 3000 rpm at 500 rpm s^−1^ acceleration and spun for 1 min. Again, layer thickness was verified *via* spectroscopic ellipsometry. The clean quartz side of the sample adhered to the base of an N-BK7, uncoated, right-angle prism (Thorlabs, PS911) with a thin layer of index matching gel (Thorlabs, G608N3, refractive index at 589.3 nm: 1.4646). The sample was exposed from below to UV light on a dimmable light stage built in-house. We used the transfer matrix method for simulations of the optical response of the system. Dispersion relations of the components were obtained from ellipsometry measurements. Thus, losses are naturally included *via* the dielectric functions.

## Results and discussion

### Dispersion relations

**Fig. 3:**
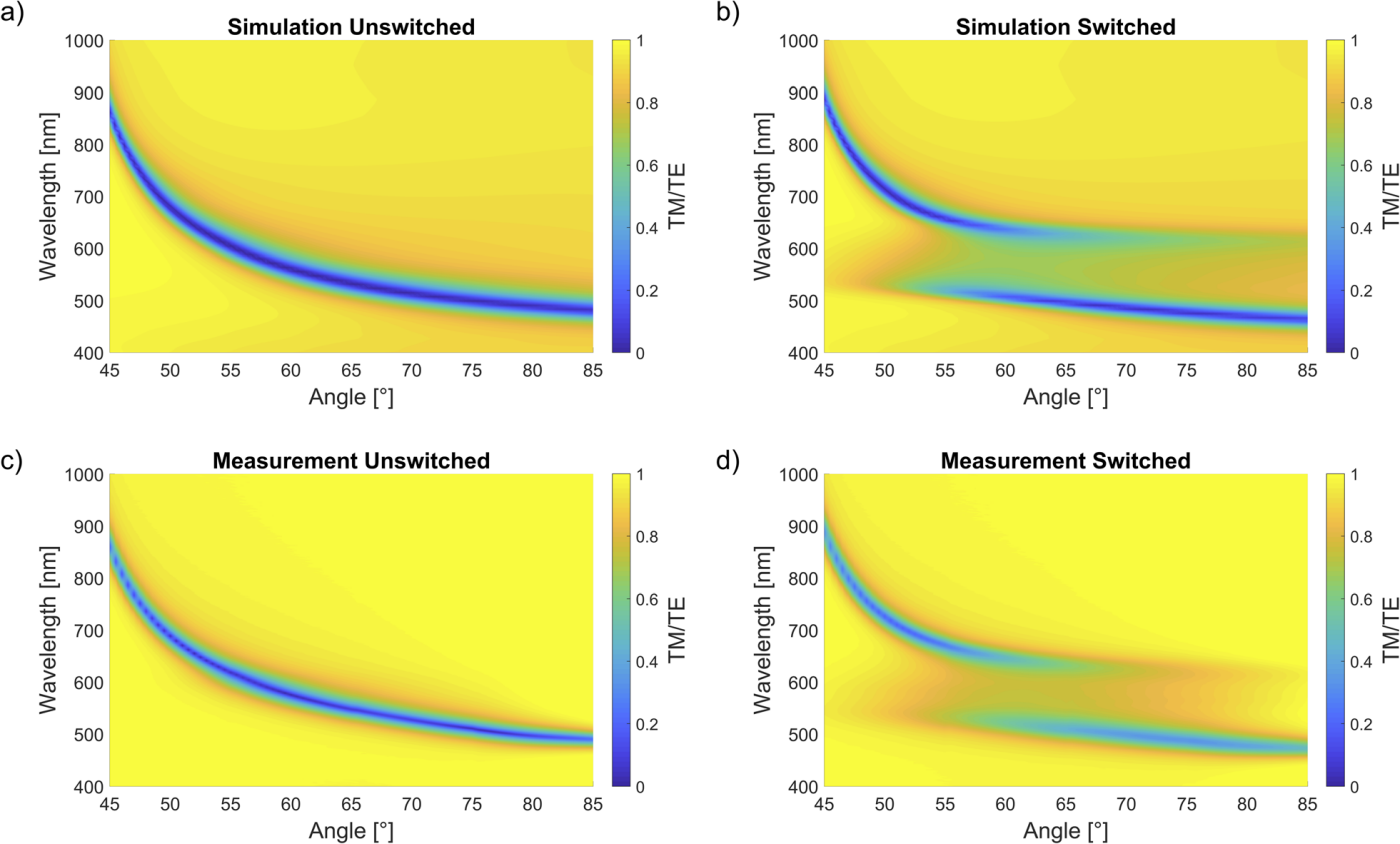
Dispersion relations for 48 nm layer of Ag covered with 36 nm layer of PS-SPO (60 wt% SPO) for (a) simulations unswitched and (b) switched, and (c) measurements unswitched and (d) switched. The false color scale shows a TM/TE scale of reflection. This normalizes the data to account for the changing light intensity due to a changing incidence angle because S-polarized light (TE) cannot couple to plasmons but p-polarized light (TM) can.

Fig. 3 shows the results of simulations and spectroscopic ellipsometer (SE) measurements. Such measurements are possible because spectroscopic ellipsometry offers the advantage of rapid measurement of the dispersion relation (see Appendix, Fig. 6) regarding backswitching during measurement. The referenced plots are displayed on a TM/TE scale of reflection, in effect showing the absorption of incident light which launches SPPs.^32^ Note, s-polarized light (TE) cannot couple to plasmons but p-polarized light (TM) can, so plotting TM/TE normalizes the data to account for the changing light intensity due to a changing incidence angle.

In the unswitched state, we observe the dispersion relation of Ag. After switching with UV light (365 nm), the enhanced dipole transitions near 600 nm of the resonating MC molecules coincide with the surface plasmon^33^ resonance of Ag. Two dispersion curves, *i.e.* the upper and lower polariton branches, appear in both the simulations and experiments which indicate and exemplify strong coupling in our straightforward system. The anticrossing occurs at the exciton energy of the photoswitched molecules. The broader and weaker experimental upper polariton branch can be attributed to several factors not included in the idealized transfer-matrix simulations. These include inhomogeneous broadening due to local chromophore concentration variations, molecular orientation disorder, surface roughness, and partial backswitching during data acquisition.

Strong coupling refers to the exchange of energy between the SPPs and the SPO molecular excitons in the adjacent dielectric layer. Indeed, the interaction of the two can be modeled as two coupled oscillators. Coupling leads to hybrid modes occurring only in the switched ON state that differ from the natural frequency of the individual, uncoupled oscillators in the OFF state where the SPO is primarily in the unswitched conformation. With irradiation of UV light of 365 nm, the proportion of SPO in the MC conformation increases and has some reflection and absorption around 600 nm, well within the range to allow spectral overlap and interference with the SPPs of the metal. The observed switching to strong coupling is a strong indicator that our system, despite the lossy nature of the involved plasmons, behaves similar to a quantum electrodynamic cavity with sufficiently low dissipation.

Another signature of cavity quantum electrodynamics is the square-root dependence of the coupling strength on *N*, the number of coupled molecules in the system. Accordingly, the number of coupled molecules can be varied here, depending on the illumination time for a set UV intensity and specific SPO concentration. An *in situ* switching video (see Appendix, Fig. 7) taken at 57° angle of incidence (AOI) for SPO 60 wt% shows active splitting of the hybrid modes with constant exposure to UV light, *i.e.* the splitting can be turned ON and tuned with the irradiation. Under carefully controlled conditions of UV illumination intensity and duration, the degree of splitting is now controllable. Controlling the extent of effective molecular switches inside the cavity *via* irradiation time or intensity allows us to tailor the coupling strength when going from the weak to the ultrastrong coupling regime, demonstrated in Fig. 3.

By controlling the magnitudes of *N* and *V*, the splitting of the hybrid modes depends on the concentration of SPO and the fraction that is switched to the MC form in the dielectric layer. As shown above, mode splitting is dependent on the square root of the concentration of emitters in the system, having its origin in the coupling of the SPP mode to the *N*-count emitters in the optical cavity volume *V*.^34,35^ Here, the chosen SPO concentration in the PS layer adjacent to the Ag thin film together with the duration and intensity of UV exposure determine the possible degree of splitting of the hybrid modes.

### Concentration dependence

**Fig. 4:**
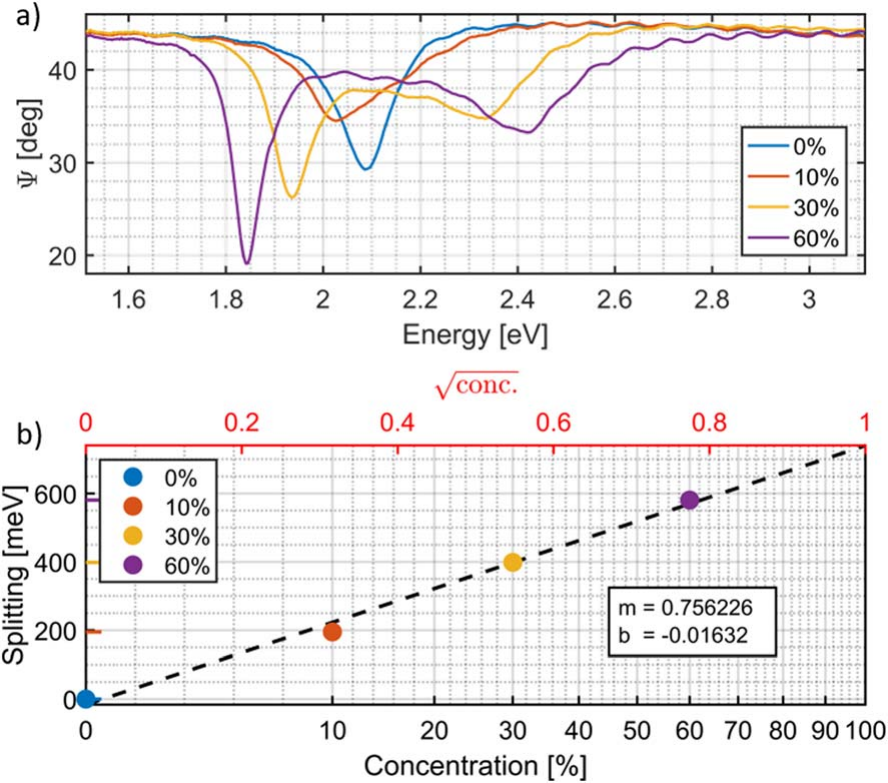
(a) Spectroscopic ellipsometry measurements of *Ψ versus* incident light energy, measured at 57° AOI. (b) Dependence of the splitting energy *vs.* the wt% of SPO. The data point at 10 wt% SPO was not measured directly because the sample displays only one peak around 2.03 eV, whereas the other peak is difficult to determine. Based on the known concentration of 10 wt% SPO, the splitting was estimated from the linear relationship of the other samples and the other peak estimated to be at 2.22 eV based on a heuristic peak fitting approach (see Appendix Fig. 8). The linear fit corresponds to the square-root dependence on concentration, while the linear concentration axis is shown for reference.

As can be seen in Fig. 4a, the fixed concentration of the SPO in the PS was varied in samples from 0 to 60 wt%. On exposure to UV light of 365 nm, *in situ* measurements at 57° angle of incidence (not shown) are used to monitor the splitting until equilibrium is reached (<30 s), after which the UV is turned off and the entire dispersion relation is measured from 45° to 85° at 0.5° steps with a sweep time of <10 min (as seen in Fig. 3d for 60 wt% SPO sample).

The greater the chosen SPO concentration in PS, the greater the resultant maximum splitting upon UV exposure of fixed intensity, in line with another study.^36^ The peak positions were determined for the 0, 30 and 60 wt% SPO samples, and the corresponding peak energy splitting calculated. Fig. 4b shows the calculated peak splitting in meV *versus* the square root of the same SPO concentration (a concentration percent axis is also shown for clarity). First, Rabi splitting of ∼600 meV for the 60 wt% SPO sample is about 29% of the molecular transition energy of 2.1 eV (or ∼14.5% when *g*/*ω* is compared, as often done in the literature), results that put the system in the regime of ultrastrong coupling. Second, a linear relationship between the splitting and square root of concentration is manifest. The 10 wt% SPO sample displays one peak around 2.025 eV, whereas the other peak is difficult to determine. Based on the known concentration of 10 wt% SPO, the splitting was estimated from the linear relationship of the other samples and the other peak estimated to be at 2.25 eV. The difficulty of accurately capturing the peaks of the 10 wt% SPO sample relate to the backswitching of the MC form of SPO. Whereas backswitching plays a relatively negligible role in the higher SPO concentrations, in the lower 10 wt% SPO concentration the backswitching and resultant peak movement play a proportionately larger role in the overall peak splitting, as evidenced when viewing in person the sample switch back over time during *in situ* measurements at 57° AOI (not shown). After the UV light is turned off, the smaller peak at higher energy melds rapidly back into the single peak like that found in the 0 wt% SPO, rendering peak identification difficult. Faster measurement at the correct angle of incidence instead of angle sweeping after switching could help to rectify the difficulty. We point out, however, that the square-root dependence of the Rabi splitting on chromophore concentration is established by the directly measured data at higher concentrations, where the splitting is clearly resolved and reproducible. The inclusion of the 10 wt% point does not define the observed trend and does not affect the conclusions drawn from it; rather, it is included for completeness.

### Time dependence

**Fig. 5:**
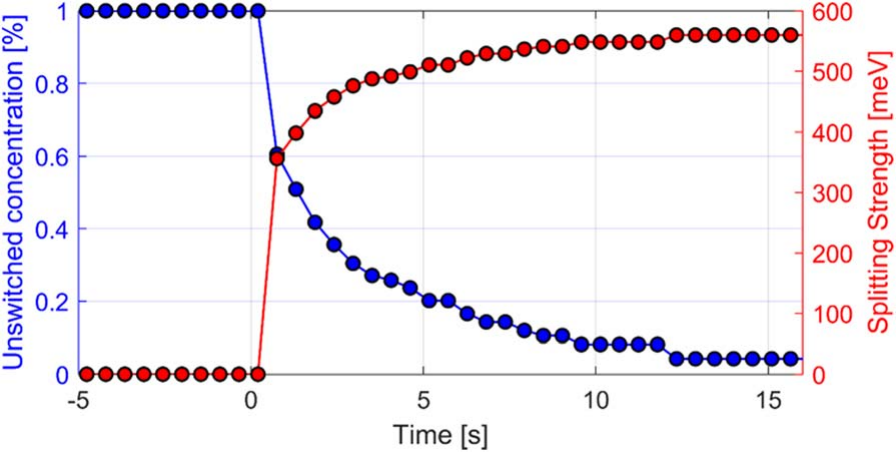
Concentration of unswitched molecules and splitting strength over time. Sample was same 60 wt% SPO used in Fig. 3c, d and 4.

In Fig. 5, we can monitor the time dependence of the switched SPO concentration and the associated degree of splitting of the hybrid modes. By determining the peak positions of the hybrid modes from recorded video (see Appendix, Fig. 7), the splitting strength of SPO molecules is shown (in red) to switch in time upon exposure to UV light (UV on at time 0). Using Fig. 4b, the percent concentration of switched SPO molecules coupled (and therefore the unswitched percentage, as plotted) can be determined (in blue). Sample was the same 60 wt% SPO used in Fig. 2c, d and 3. Linear dependence of splitting on √*N* proves that the strength of energy splitting is in fact based on quantum yield of the switched molecules and can be controlled by the irradiation time. Switching and all-optical control of the degree of splitting is thus demonstrated.

The SPO chromophore was chosen to demonstrate the principle of light-controlled tuning of the coupling strength, rather than to optimize long-term cyclability. Reversible switching and fatigue behavior in related photochromic plasmonic systems have been demonstrated previously, particularly in work from Elbahri's group.^21,22,29,30^

The strength of the coupling between matter and light that has been shown here to be controlled by the concentration of and conformation change in photoswitchable molecules (thereby changing their dipole moment and by decreasing the optical volume *via* the real part of the refractive index) relies on an important parameter, namely *N*, the number of coupled molecules in the cavity. *N* relates to the quantum yield of the photosensitive molecules in the cavity. The coupling strength is directly proportional to √*N* and is essential in tailoring the magnitude of Rabi splitting. Considering the light–matter interactions,^37^ we illustrate the role of √*N* in tailoring the coupling strength by controlling the irradiation time. Indeed, as can be seen in Fig. 5 and the SI Video S1, the splitting can be turned on by UV light exposure. Under carefully controlled conditions of UV illumination intensity and duration, the degree of splitting is controllable. Investigations with suitable light sources should delve more deeply into this promising aspect of controlling Rabi splitting dynamically. Any possible UV degradation of the organics involved could be tackled by consulting the multitude of chromophoric molecules already known to chemists.

Thus, our work directly demonstrates that the molecular properties themselves determine the splitting. By varying the filling factor of photoswitchable molecules, we systematically tune the real part of the refractive index (*n*), which shifts the cavity mode position and reduces the effective cavity mode volume *V*. This enhanced confinement directly increases the coupling strength, while the imaginary part (*k*) modulates only absorption magnitude and does not affect the mode position.

We define this as a volume-driven cavity mode effect because the splitting and mode shift are controlled primarily by *n*, in combination with the evanescent confinement of the plasmonic field. TM simulations at the highest filling factor confirm this effect. The experimental results show square root scaling of the splitting with filling factor, and *in situ* temporal measurements (<30 s under UV exposure) demonstrate the dynamics of switching. Thus, our study establishes a mechanistic link: molecular properties → *n* → mode volume → splitting.

## Conclusions

In summary, we have demonstrated that, despite the lossy nature of plasmonic systems, ultrastrong light-controlled molecular plasmonic coupling can be obtained in a simple Kretschmann configuration at the interface between a metal and a chromophore containing polymer. Photoswitching is reversible, and the coupling strength can be easily controlled by the light intensity as well as the concentration of the chromophore in the polymer matrix. It could be promising to investigate the extension of our approach to other advanced polaritonic material systems, specifically to the newly emerging 2D materials.^38^

## Author contributions

B. S. and R.-M. F. designed, constructed, executed and wrote about experiments as well as conceptualized and coded simulations. T. S. provided background chemistry knowledge. M. E. conceptualized the underlying scientific questions with the real-part-driven elucidation of the cavity mode volume dependence, the temporal design, and oversaw the writing of the introduction and conclusions, bringing the results forward within the literature framework. F. F. supervised the work of B. S. and R.-M. F. F. F., M. E., and T. S. were principle investigators in the abovementioned Project C1 within Collaborative Research Center 677.

## Conflicts of interest

There are no conflicts to declare.

## Appendix A

**Fig. 6:**
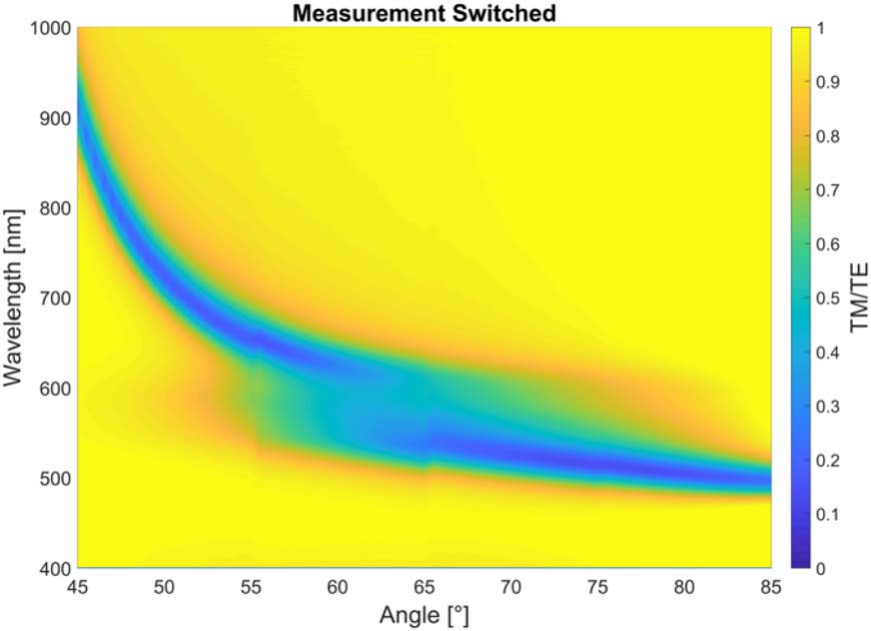
Extent of backswitching investigated. Using the sample from Fig. 3 (48 nm layer of Ag covered with 36 nm layer of PS-SPO (60 wt% SPO)), immediate measurements were made from 55–65° (duration: ∼90 s) after freshly switching with UV light, the results of which were superimposed on Fig. 3d to show the extent of backswitching taking place during the original 45–85° measurements. A small amount of backswitching can be seen to have taken place during the original measurements, which had taken about 10 minutes.

**Fig. 7:**
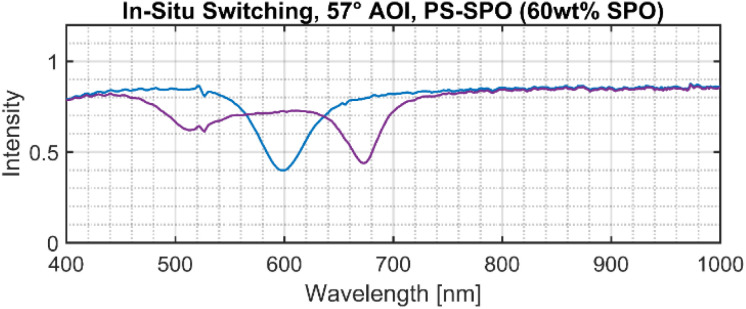
Picture from video of *in situ* splitting of PS-SPO (60 wt% SPO) on Ag in a Kretschmann configuration. Splitting begins when the single peak begins to divide and corresponds to the UV light (365 nm) being turned on. After being exposed for 16 s, the splitting of the peaks stabilizes as equilibrium is reached. The video provided as SI is sped up to make viewing more convenient.

**Fig. 8:**
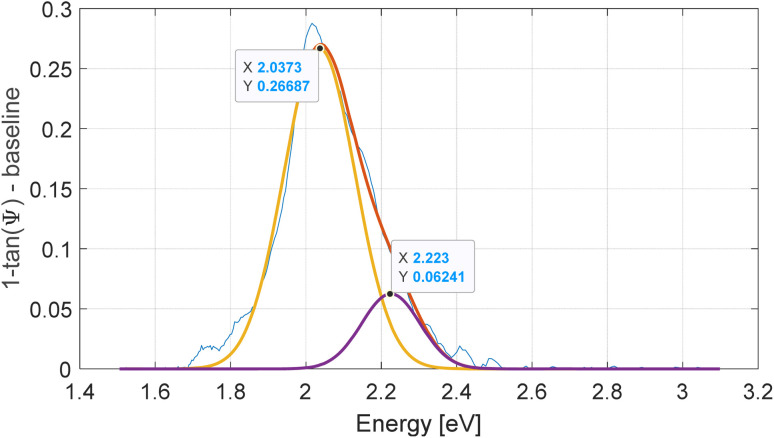
Peak fitting using two Gaussians to determine the peak splitting of the 10% concentration in Fig. 4.

## Data Availability

Data for this article, including description of data types are available at opendata@uni-kiel at https://opendata.uni-kiel.de/receive/fdr_mods_00000323?accesskey=JgWf4g107UweiKyhMPBJEUVLv9mFY4TT. Supplementary information (SI): Video S1: InSituSwitching.avi. See DOI: https://doi.org/10.1039/d5na01061f.
